# Substance Abuse Treatment, HIV/AIDS, and the Continuum of Response for People Who Inject Drugs

**DOI:** 10.1155/2012/541489

**Published:** 2012-11-29

**Authors:** Thomas F. Kresina, Robert Lubran, H. Westley Clark, Laura W. Cheever

**Affiliations:** ^1^Division of Pharmacologic Therapies, Center for Substance Abuse Treatment, Substance Abuse and Mental Health Services Administration, Rockville, MD 20857, USA; ^2^HIV/AIDS Bureau, Health Resources and Services Administration, Rockville, MD 20857, USA

## Abstract

The continuum of response (CoR) to HIV/AIDS is a framework for implementation of HIV prevention, care, and treatment programs based on a national strategic plan for HIV/AIDS services. The CoR for people who inject drugs (PWID) is an important extension of the developed CoR to HIV/AIDS. The CoR-PWID employs stakeholders who together plan, develop, pilot, and provide a full range of services that address the various prevention, care/support, and treatment needs of people, families, and communities infected or affected by HIV/AIDS and injection drug use. The CoR-PWID comprises a broad range of services that include but are not limited to the World Health Organization priority interventions for HIV/AIDS prevention, treatment, and care in the health sector and the package of essential interventions for the prevention, treatment, and care of HIV for people who inject drugs. Implementation of these well-defined, essential prevention, care/support, and treatment services, in addition to locally defined needed services, in a coordinated fashion is important to clients, their families, and communities. The CoR-PWID is, therefore, a necessary framework essential for service development for countries that address HIV/AIDS in populations of PWID.

## 1. The CoR to HIV/AIDS

**Figure 1:**
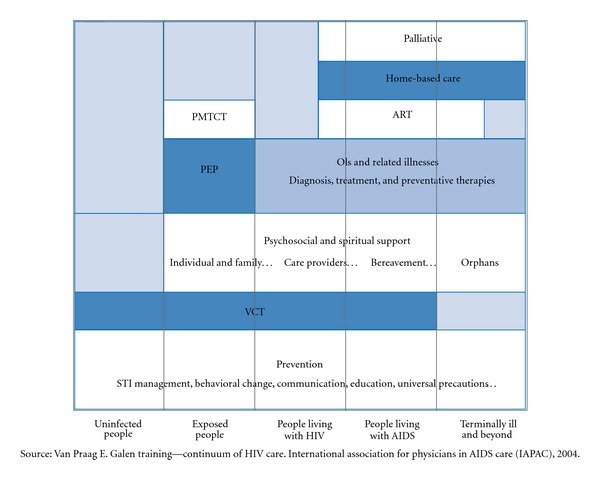
Continuum for a comprehensive response to HIV/AIDS.

HIV/AIDS is a complex disease that results in complex needs from patients infected with the virus, their partners, extended family members, and communities [1]. The concept of a continuum of care to provide for the needs of patients, family members, and communities affected by HIV/AIDS was developed early in the epidemic [1]. A comprehensive HIV/AIDS care continuum framework was proposed and consisted of patient discharge planning and referrals among health facilities treating patients with HIV/AIDS and services that included voluntary counseling and testing, community-based services, blood transfusion services, self-help groups, and home care. The care continuum for HIV/AIDS was expanded to encompass the needs of care providers including home care providers [2]. The expansion proposed an international care agenda that included policy strategies that focused on the caregiver where a range of public, private, and no-governmental sectors would come together with the common purpose of insuring that households affected by HIV/AIDS are protected and supported to ensure survival. The CoR to HIV/AIDS builds on these previous models by developing a framework that strengthens national leadership while delivering improved broader health outcomes [3]. This new paradigm supports country ownership and enhances a sustainable national AIDS response to the epidemic. The paradigm has stakeholders planning and providing a full range of services to address the various prevention, care/support, and treatment needs of people, families, and communities infected or affected by HIV/AIDS. The goal is to maximize health outcomes through approaches that decrease HIV transmission, slow disease progression and improve the sense of wellbeing at the individual, family, and community levels. This goal is obtainable as shown by the CoR for HIV/AIDS developed in the United States through the Ryan White HIV/AIDS program. This program creates a true medical home for patients with HIV/AIDS through the support of a coordinated team of health care providers delivering high-quality medical care as well as psychological/psychiatric services, substance abuse treatment, adherence counseling, and social services [4]. Thus, the CoR for HIV/AIDS comprises well-defined essential prevention, care/support, and treatment services (shown in Figure 1), in addition to locally defined needed services. These services when provided in a coordinated fashion to clients, family members, and communities are highly effective [5]. Effective prevention, care/support, and treatment services included in the CoR for HIV/AIDS are identified based on (a) community needs and on epidemiologic data, (b) strategies defined locally, (c) assessed individual needs, and (d) the needs that change over time or life-span approaches.

At the individual level, the CoR results in the government orchestrating a health system that identifies populations at-risk and maintains them in appropriate programs that provide services to address identified needs through the lifespan. Prevention services are available for those at-risk for HIV infection and care and treatment services are provided if the individual becomes HIV infected. The CoR also provides for their non-HIV needs through the provision of social, legal, and other wrap around services insuring their ability to access HIV prevention, care, and treatment services. 

At the family level, family engagement can facilitate HIV prevention, care, and treatment. The use of patient and family advisors results in improved health outcomes including decreased length of stay in inpatient facilities and higher patient satisfaction [6]. Family-centered care can improve access to and retention in care, particularly for women and children in maternity scenarios where collaborative care and health care integration are successful and sustainable practices [7]. 

At the community level, engagement of PWID and are HIV infected in the planning and delivery of services in the CoR can improve service delivery through the determination of need, prioritization of service, assessment of quality of services and elimination of barriers to accessing services. Alternatively, engagement of the community to address HIV/AIDS through public education and community mobilization on community health can result in adolescents increasing their knowledge base of health risks and communication on health issues and adults increasing their understanding of adolescent issues related to HIV/AIDS [7, 8].

Through the development of a CoR, countries can provide a comprehensive system of prevention, care, and support to meets the health needs of their people. As the US Global Health Initiative recognizes, the CoR is needed, not only for particular diseases such as HIV, but also for the whole range of public health issues including injection drug use.

## 2. The CoR to HIV/AIDS for PWID

The CoR-PWID is a service platform for a national response to an HIV/AIDS epidemic. While there are national HIV/AIDS epidemics that are mainly limited to key populations such as injection drug users, for example the HIV/AIDS in Vietnam, diffusion of HIV infection from key populations to the general population through various social constructs has been documented [9]. Therefore, virtually every country can benefit from the development of a CoR-PWID as part of its national HIV/AIDS strategy. The combination prevention program included in the CoR-PWID improves access to the health system for PWID thereby improving their health outcomes. In addition, the CoR-PWID promotes the health integration of the prevention services as well as sustainability of the combination prevention services. The result is a set of combination prevention services that thoroughly and strategically address the risk of HIV transmission and acquisition for key populations. When fully developed and implemented, the CoR-PWID provides a continuum of linked prevention, care, and treatment services for people who inject drugs. Combination prevention programs for PWID are based on the premise that no single intervention is fully efficacious in the prevention of HIV transmission and its acquisition. Rather, they are the set of optimal biomedical, behavioral, and structural interventions, of high quality, implemented at a national scale and targeting key populations that impact the HIV epidemic for key populations.

**Case Study**

(A model for retention and continuity of care and treatment for opioid-dependent injection drug users.) A nongovernment organization (NGO) worked collaboratively with the National Government to develop and implement a model program of retention and continuity of care for opioid dependence and HIV infection. The model utilizes the National Detoxification Service, State AIDS Centers, NGO managed opioid treatment and rehabilitation centers, NGO outreach programs, and the community. The model program was developed and implemented as a pilot demonstration project to provide essential health services to injection drug users and retain them in care. The interventions developed and implemented comprised HIV testing and counseling, HIV/AIDS opioid dependence postgraduate curriculum for care providers, peer support groups, opioid detoxification, and treatment follow-up phone monitoring, women's opioid dependence support services and short messaging services (SMS) for targeting injection drug users. These services and interventions promoted the integration and utilization of HIV/AIDS health services and opioid treatment services to form an evidence-based health service delivery model providing essential services to PWID and people living with HIV/AIDS [10].

Combination prevention interventions, as shown in the case study above, comprise a broad range of services that include specific targeted interventions addressing both injection drug use and HIV infection. Such interventions are articulated in the WHO priority interventions for HIV/AIDS prevention, treatment and care in the health sector [11], and the WHO, UNODC, and UNAIDS the package of essential interventions for the prevention, treatment and care of HIV for people who inject drugs [12]. These well-defined essential prevention, care/support, and treatment services, in addition to locally defined services are important to clients, family members, and communities. These evidence-based interventions, shown in Table 1, require four important characteristics as part of their implementation in a CoR-PWID to maximize effectiveness. These interventions need to be (a) part of a public health policy, (b) human rights-based, (c) gender responsive, and (d) community owned. These essential interventions integrated into the set of priority interventions for HIV/AIDS prevention, care, and treatment in the health sector (Table 1) form the basis of a national framework for the set of interventions and service comprising the CoR-PWID.

As noted earlier, no single intervention will prevent or reverse the growing national HIV epidemics due to injection drug use and abuse. The greatest impact will be obtained when the interventions are provided through an integrated services platform in a comprehensive high-quality fashion that is scaled nationally. And in order to reach all of those seeking HIV prevention, care, and treatment services, health service platforms need to provide an enabling environment that establishes confidentiality. Also, programs need to develop patient-provider trusting relationships. Both community outreach and peer-to-peer services can promote full service utilization. If the national Ministries of Health embrace and support these health services and interventions through a supportive legal and policy framework, the CoR-PWID will be validated as part of the national public health strategy to address the HIV/AIDS epidemic.

## 3. Substance Abuse Treatment as HIV ****Prevention and Part of the CoR-PWID

People who inject drugs face multiple health and social risks from injection practices as well as the lifestyle of drug use and abuse [13, 14]. Injection practices, which include unsterile injection practices, contaminated drug paraphernalia, and drug adulterants, enhance the risk of drug overdose, infections from bacterial, fungal, and protozoal pathogens and parenterally acquired viral infections, including HIV and hepatitis [15]. Lifestyle events, such as homelessness, poverty, mental illness, or family abandonment, as well as lifestyle behaviors, such as multiple sexual partners or criminal behavior, increase the risk of sexually transmitted infections and comorbidities. The medical cooccurring conditions are specifically prevalent in key populations, especially PWID. Estimates for the population of PWID are available for at least 130 countries with approximately 78% of the 13.2 million people who inject drugs living in developing or transitional countries [16]. 

Forty-one countries have reported a high prevalence (>5%) of HIV infection in key populations (PWID, sex workers, and men having sex with men). Globally, PWID now account for at least 10% of all new HIV infections which are estimated at 5 million per year [17]. In chronic HIV infection, AIDS has been reported as the leading cause of death in PWID [18]. Epidemiological data of HIV infection show that generalized HIV epidemics can result from diffusion transmission of HIV from key populations. Thus, it is important for countries and regions, as part of the CoR-PWID, to undertake surveillance studies to identify current drug use patterns and develop the best practices for the treatment of individuals who inject and abuse illicit drugs. 

Drug injection can rapidly develop into drug dependence, a chronic, relapsing neurophysiological disease resulting from the prolonged physiological effects of drug(s) acting in the brain. The neurochemical abnormalities occurring in the brain that result from chronic use and drug injection are the underlying cause of many of the observed physical and behavioral aspects of abuse and dependence. The brain abnormalities associated with addiction are wide ranging, complex, and long lasting [19–21]. They can involve abnormal brain signaling pathways, psychological conditioning or stress, and social factors that result in drug cravings leading to a predisposition to relapse even months or years after drug(s) use cessation. Thus, substance abuse/dependence can be most effectively addressed in a multifaceted medical-based paradigm to address the complex changes in the brain along with other comorbidities. The medical-based paradigm comprises a comprehensive program of interventions delivered through the course of long-term treatment. Comprehensive treatment programs include behavioral, social rehabilitative components, and biological (pharmacological) components comprising a continuum of care, as shown in Table 2. Behavioral therapy interventions have been extensively researched and are critical components of the treatment of all drug addictions. Social rehabilitative components are also important as an integral element of a treatment environment and as a wrap-around service.

The use of medications, as part of comprehensive substance abuse treatment, is particularly important for PWID and who abuse opioids or who are opioid dependent [12]. Globally the most common medication used for the treatment of opioid dependence is methadone [22]. Methadone is an opioid agonist whose use in treatment and research is controlled by international conventions. The international conventions allow for differing levels of regulation for individual countries that utilize methadone. Thus, in a highly regulated and structured environment, as in the United States, methadone is dispensed daily at Opioid Treatment Programs (OTPs). These OTPs are increasingly providing wrap-around services to address important patient needs, enhance time in treatment, and promote recovery. Alternatively, methadone can be provided to patients in treatment through prescription or through specific regulated pharmacies.

An alternative medication to methadone is buprenorphine, a partial opioid agonist. Buprenorphine, while regulated, can be prescribed in a primary health care setting even in a highly regulated and structured environment. Thus, opioid dependence treatment can be accessed and provided similar to other illnesses with the result being reduced stigma/discrimination. As part of the CoR-PWID, both medications can be a component of a substance abuse treatment programs in an effort to address the reduced quality of life as well as reduced physical and mental functioning commonly found in drug injectors and drug abuse/dependence [23]. 

Naltrexone is a nonnarcotic prescription medication for use in relapse prevention to opioid use. Unlike methadone, there is no negative reinforcement (opioid withdrawal) upon discontinuation. Naltrexone is most effective when utilized subsequent to the medical detoxification from opioids. The effectiveness of naltrexone treatment depends upon patient motivation and a social support system that promotes medication adherence [24]. 

Depot-naltrexone (Vivitrol) addresses the reduced medication adherence of oral naltrexone through a monthly injectable formulation. Increased medication adherence was shown in a recent Phase 3 clinical trial that confirmed Vivitrol's safety and efficacy in the prevention of relapse to heroin use in a cohort of injection drug users [25]. Currently, studies are underway to determine the most efficacious service model(s) for the use of depot-naltrexone in the treatment of relapse prevention to heroin use and as part of the CoR for opioid users.

Until recently, the global availability and consumption of opioid agonists, such as methadone and buprenorphine, as well as opioid antagonists, such as naltrexone, have been below the levels needed for international research to demonstrate local efficacy and to develop local evidence-based best medical practices [26, 27]. In addition, the global availability and use of methadone has not been sufficient to implement the well-documented efficacy shown for the treatment of opioid dependence that has been developed by research over the last 40 years [27]. However, a strong research base in Western countries has resulted in the development of evidence-based medical practices using opioid agonists in maintenance treatment regimens and opioid antagonists in relapse prevention strategies. The recognition of these evidence-based medical treatment for opioid abuse and dependence has resulted in a substantial global increase in the medical use of opioid agonists and psychotropic medications to address opioid dependence [28–30]. This increase is particularly evident in the initiation of new pharmacotherapy programs in regions of Europe, North America, Africa, Asia, and Oceania. While in other regions of the world, the medical use of opioid agonists and psychotropic medications have not substantially increased; the implementation of pharmacotherapy programs have begun as part of the global effort to reduce HIV/AIDS. These HIV prevention programs have utilized medications as an element of programs that target injection drug users to reduce their risk of both acquiring and transmitting HIV infection. Thus, the recent international expansion of the use of pharmacotherapy for opioid dependence as a result of efforts to increase access and availability of evidence-based treatments for opioid dependence as well as efforts to reduce the spread of infectious diseases, such as HIV/AIDS, a life priority of PWID.

Addressing the life priorities of opioid users in the CoR-PWID is important to enhance the quality of life of the patient in treatment, promote treatment acceptance, and further develop the trusting patient-provider relationship. Life priorities for opioid users have been reported as concerns about HIV and treatment of infection with HIV, housing, money, and protection from violence [31]. The CoR-PWID with its integrated approach to services for HIV/AIDS and injection drug use is centrally positioned to address the life priorities of opioid users. 

Substance abuse is a complex medical disorder composed of multiple physiologic, social, and behavioral problems often interrelated with psychological illness. As part of the CoR-PWID, health care providers need to screen people who inject drugs for psychological illness as well as associated trauma and abuse [32]. Although PWID may be self-medicating due to a history of trauma or abuse, an initial focus on the medical treatment of drug abuse is often necessary to create sufficient patient stability from which other treatments can begin. Patient stability is further increased with gender-based, trauma-informed care, and treatment coupled to both mental health services and substance abuse treatment, thereby enhancing the medical outcomes of treatment for other comorbidities [33]. An effective treatment strategy for PWID is to match a comprehensive treatment plan to the individual's particular substance abuse problems and needs. Desired treatment outcomes should (a) reduce dependence on drugs of abuse, (b) reduce morbidity and mortality of and associated with drugs of abuse, and (c) maximize the patients' abilities to access services and achieve social integration.

## 4. HIV Prevention in the CoR-PWID: ****Integrating Medication-Assisted Treatment ****into HIV Prevention Services

Medication-assisted treatment or MAT is the use of medications coupled to behavioral therapies as part of a comprehensive substance abuse treatment program [34]. MAT has three main stages or phases of treatment: induction, or the introduction of medications into the patients treatment program; stabilization, or the determination of an appropriate dose of medication for patient stabilization; and maintenance, or long-term administration of the stabilizing dose. After long-term treatment the patient may elect to end MAT as part of their long-term treatment program. So an additional stage/phase can be the titer or ending of medication treatment. PWID and who are opioid dependent travel through the three stages of treatment, sometimes linearly and sometimes with oscillations between phases. The ultimate goal upon entering MAT is a good clinical outcome, which includes the recovery from opioid abuse and dependence and the social reintegration back into society. The individual in recovery is a participating in the CoR-PWID to the fullest extent as a functioning member of the community contributing to the social fiber and health of the community. Thus, a fundamental component of the CoR-PWID and a foundation of MAT are the obtainment of recovery from opioid abuse and dependence [34]. 

Established MAT programs can frequently be found as a stand-alone service with limited to none prevention services integrated into the treatment program. However, there are important CoR HIV prevention interventions for PWID and their integration into MAT programs is important as HIV prevention interventions. They are the provision of clean needles and syringes through syringe service programs and associated HIV testing and counseling programs. These HIV prevention interventions, when integrated into MAT programs, maximize the enrollment in MAT programs and thereby maximize HIV prevention efforts [35, 36]. Maximizing HIV prevention efforts targeting PWID and those dependent on opioids are critical to prevent HIV infection in key populations.

Integrating drug abuse treatment and early HIV prevention interventions, particularly HIV testing and counseling, are important as CoR-PWID components of the newly emerging Seek, Test, Treat and Retain strategy [37, 38]. This is an engagement and retention strategy that outreach workers can employ with PWID to reduce their risk for HIV infection. Outreach workers can be employed to seek out PWID, establish their HIV status through HIV testing, counsel sexual risk reduction, and thereby address HIV risk behaviors with a subsequent emphasis on treatment for their substance use disorder. 

 As noted earlier, there is not significant integration of HIV testing and counseling in MAT as part of the CoR-PWID. In the USA, while approximately 90% of opioid treatment programs provide some form of federally mandated HIV/AIDS education, only 74% of opioid treatment programs offered HIV testing [39]. These services appear underutilized in that approximately one-in-three persons receiving substance abuse treatment also received HIV testing and counselling [40]. Globally, although substantial efforts are being made to increase the availability of HIV testing, key populations remain underserved with regard to HIV prevention services. It is estimated that only 10% of persons at-risk for HIV infection receive HIV testing. Thus, strategies such as opt-out testing, home-based testing, door-to-door testing, and providing dedicated HIV testings counselors at point-of-service locations are being utilized as part of the CoR-PWID. Studies have shown that key populations prefer point-of-service HIV testing, however, this intervention requires additional measures to support HIV-positive individuals entering into HIV care and treatment [41]. 

## 5. HIV Transmission Prevention in CoR-PWID: Integration of MAT into HIV Care and ****Treatment

A significant factor in not reducing the global HIV epidemic is the lack of entrance into HIV care and treatment by key populations. These populations, which include PWID, encounter numerous barriers in accessing HIV care and treatment. In addition, once in treatment these individuals often suffer stigma and discrimination as they receive their needed medical care. The result is an increase in the prevalence of medical and psychiatric comorbidities for PWID, as well as social issues and high-risk behaviors. PWID also have the worse clinical outcomes with a higher mortality rate compared to the nondrug using populations infected with HIV [42]. 

The increased mortality rate noted in PWID is related to their late presentation for HIV care. Patients who present late for care and treatment of HIV/AIDS are at a higher risk of significant clinical complications and are thus more difficult to clinically manage. Late presentation for treatment of HIV/AIDS is a common scenario leading to death [43]. A recent study has documented a highly lethal neurological syndrome found in HIV-infected drug abusers [44]. Although rare, the newly described syndrome is highly lethal with a mean survival time of 21 days after diagnosis. The authors suggest that access and initiation of antiretroviral therapy may provide a better outcome for these patients. In addition, substance abuse treatment, particularly MAT, which has been shown to enhance the health status and quality of life of PWID, as well as reduce mortality, would be an important adjunct to antiretroviral treatment for these patients. Thus, as noted earlier, integrating both MAT with antiretroviral treatment in a HIV primary care setting is important to optimize the CoR-PWID.

How MAT can be integrated in HIV primary care programs depends on the country's regulatory framework. In the United States, medications except methadone, can be prescribed to patients in a HIV primary care or outpatient HIV clinical care setting. The federal regulations in the United States require methadone to be dispenses in OTPs. However, in this setting studies have shown that HIV care and antiretroviral treatment can be effectively prescribed either as directly observed therapy or as routine care. Other countries, such as Australia, have less stringent federal regulations for prescribing controlled medications and all medications comprising MAT can be provided in a primary care setting. In either case, the important aspect of providing integrated MAT and HIV primary care is the single location/clinic. In that case, the patient can receive all the needed services to support their recovery from drug dependence as well as care and treatment for HIV infection. 

## 6. Conclusion 

The CoR-PWID is an essential tool in the HIV prevention toolbox of national HIV/AIDS prevention, care, and treatment strategies that address concentrated HIV epidemics related to injection drug use. Implementation at a national level of these well-defined, essential prevention, care/support, and treatment services, in particular MAT for opioid dependence, is fundamental for the CoR-PWID. The addition of these services to locally defined services results in the prevention of incident infections and HIV transmission, through the development of important health services for people living with HIV, their families, and communities.

## Figures and Tables

**Table 1:** Listing of internationally accepted essential interventions for HIV prevention for people who use drugs.

(1) Needle and syringe programmes (NSP)/syringe service programs (SSP)	
(2) Substance use disorder treatment, including medication-assisted treatment (MAT) and opioid substitution treatment (OST)	
(3) HIV counselling and testing (HCT)	
(4) Antiretroviral therapy (ART)	
(5) Prevention and treatment of sexually transmitted infection (STI)	
(6) Condom distribution programs for PWID and their sex partners	
(7) Targeted information, education, and communication (IEC) for PWID and their sex partners	
(8) Vaccination, diagnosis, and treatment of viral hepatitis infection	
(9) Prevention, diagnosis, and treatment of tuberculosis	

**Table 2:** Elements of the continuum of care for people who use drugs, abuse, or are drug dependent.

(1) Prevention of drug initiation	
Individual targeted interventions through the lifespan	
Family-targeted interventions	
Community interventions	

(2) Identification of substane use conditions	
Screening for drug use	
Case finding	
Assessment and diagnosis	

(3) Initiation and engagement in drug treatment	
Brief intervention	
Promoting engagement, case management/care navigators	
Detoxification/withdrawal management	
Assessment of social, comorbid medical conditions, and cooccurring disorders	

(4) Long-term treatment of substance use illness	
Psychosocial	
Pharmacotherapy	
Treatment of comorbid medical conditions and cooccurring disorders	
Promotion of treatment engagement and social stability through legal, social, educational, financial support	

(5) Primary care and posttreatment management of patient	
Recovery	
Medical home	
Relapse prevention	
Rehabilitation	
